# RNA editing in response to COVID-19 vaccines: unveiling dynamic epigenetic regulation of host immunity

**DOI:** 10.3389/fimmu.2024.1413704

**Published:** 2024-09-06

**Authors:** Yun-Yun Jin, Ya-Ping Liang, Jia-Qi Pan, Wen-Hao Huang, Yan-Meng Feng, Wei-Jia Sui, Han Yu, Xiao-Dan Tang, Lin Zhu, Jian-Huan Chen

**Affiliations:** ^1^ Laboratory of Genomic and Precision Medicine, Wuxi School of Medicine, Jiangnan University, Wuxi, China; ^2^ Joint Primate Research Center for Chronic Diseases, Institute of Zoology of Guangdong Academy of Science, Jiangnan University, Wuxi, China; ^3^ Jiangnan University Brain Institute, Jiangnan University, Wuxi, Jiangsu, China

**Keywords:** SARS-CoV-2, A-to-I RNA editing, ADARs, COVID-19 vaccine, immune

## Abstract

**Background:**

COVID-19 vaccines are crucial for reducing the threat and burden of the pandemic on global public health, yet the epigenetic, especially RNA editing in response to the vaccines remains unelucidated.

**Results:**

Our current study performed an epitranscriptomic analysis of RNA-Seq data of 260 blood samples from 102 healthy and SARS-CoV-2 naïve individuals receiving different doses of the COVID-19 vaccine and revealed dynamic, transcriptome-wide adenosine to inosine (A-to-I) RNA editing changes in response to COVID-19 vaccines (RNA editing in response to COVID-19 vaccines). 5592 differential RNA editing (DRE) sites in 1820 genes were identified, with most of them showing up-regulated RNA editing and correlated with increased expression of edited genes. These deferentially edited genes were primarily involved in immune- and virus-related gene functions and pathways. Differential *ADAR* expression probably contributed to RNA editing in response to COVID-19 vaccines. One of the most significant DRE in RNA editing in response to COVID-19 vaccines was in apolipoprotein L6 (*APOL6*) 3’ UTR, which positively correlated with its up-regulated expression. In addition, recoded key antiviral and immune-related proteins such as IFI30 and GBP1 recoded by missense editing was observed as an essential component of RNA editing in response to COVID-19 vaccines. Furthermore, both RNA editing in response to COVID-19 vaccines and its functions dynamically depended on the number of vaccine doses.

**Conclusion:**

Our results thus underscored the potential impact of blood RNA editing in response to COVID-19 vaccines on the host’s molecular immune system.

## Introduction

SARS-CoV-2 emerged in late 2019 and caused a global pandemic of coronavirus disease 2019 (COVID-19), posing serious threats to human health and society (1). COVID-19 vaccines are crucial to prevent severe disease and death caused by SARS-CoV-2. Vaccines could induce a robust immune response, triggering the production of antibodies and memory cells that can recognize and act on the virus (2, 3). However, COVID-19 vaccines could also pose immune-related challenges, such as the risk of allergic reactions (4, 5), vaccine breakthrough infections (6, 7), and waning immunity over time (8, 9). These challenges require further research and monitoring to ensure the safety and efficacy of the vaccines. Additionally, individuals with different immune conditions may respond differently to COVID-19 vaccines. The underlying regulatory mechanism involved in the immune response to COVID-19 vaccines requires further investigation.

Adenines to inosine (A-to-I) RNA editing, mediated by adenosine deaminase acting on RNA (ADARs), is one of the most widespread post-transcriptional epigenetic RNA modifications (10) and is recognized as A-to-G transitions during translation (11, 12). A-to-I RNA editing and ADARs play a key role in host antiviral activities by acting on the negative-strand RNA of SARS-CoV-2 during the viral replication (1, 13–17). Additionally, emerging studies have shown that SARS-CoV-2 infection changes the A-to-I RNA editing level, which could further affect the evolution of SARS-CoV-2 and the immune response (18–20). Nevertheless, it remains to be elucidated whether and how RNA editing plays a role in the immune response to COVID-19 vaccines.

In the current study, we performed a transcriptome-wide analysis of the blood A-to-I RNA editing profiles of individuals vaccinated with COVID-19 vaccines to investigate RNA editing response to COVID-19 vaccines (RNA editing in response to COVID-19 vaccines). By identifying substantial RNA editing in response to COVID-19 vaccines, our results linked A-to-I RNA editing to the immune response to COVID-19 vaccines.

## Materials and methods

### RNA-Seq reads download and processing

To explore RNA editing in response to COVID-19 vaccines, RNA-Seq raw data was downloaded from the European Nucleotide Archive (ENA) (https://www.ebi.ac.uk/ena). The dataset (PRJNA821445) contained 260 blood samples from 102 adults who received one, two, and three doses of COVID-19 vaccines (2).

By using a pipeline described in our previous study, the FASTQC was used for quality control of sequencing reads (21). Reads that passed quality control were mapped to the human genome (UCSC hg38) using RNA STAR (version 2.7.0e) (22). Then, the output BAM files were filtered by SAMtools (version 1.9) to remove multi-mapped and duplicated reads (23). Finally, GATK (version 4.1.3) was used to conduct base quality score recalibration (24).

### RNA editing analysis

Single nucleotide variants (SNVs) were called from the BAM files using VarScan (version 2.4.3) (25), and annotated using the Ensembl Variant Effect Predictor (VEP) (26). Only variants that met the following criteria were retained: base quality ≥ 25, total sequencing depth ≥ 10, alternative allele depth ≥ 2, and alternative allele frequency (AAF) ≥ 1%. SNVs that met the following criteria were removed unless annotated as known RNA editing sites in the REDIportal V2.0 database (27): 1) located in homopolymer runs ≥ 5 nucleotides (nt) or simple repeats; 2) located in mitochondrial DNA; 3) located within 6 nt from splice junctions; 4) located within 1 nt from RNA insertion-deletion (INDEL); 5) within 4% to the ends of reads; 6) annotated as known variants in the dbSNP database Build 142; 7) more than 90% of samples had an AAF equal to 100% or between 40% and 60%; with an editing level < 5% in both the control and COVID-19 vaccines groups.

### Gene expression quantification

The Rsubread package of the R language was used to calculate pseudo counts of the RNA expression (28) and normalized gene expression levels (transcript per million, TPM).

### Protein structure prediction of missense RNA editing variants

Protein structure prediction was performed using the DDMut online tool (https://biosig.lab.uq.edu.au/ddmut/) to evaluate the impact of the missense changes on the edited protein (29).

### Prediction of RNA secondary structure

RNA secondary structure prediction was performed using the RNAfold Web Server online tool (http://rna.tbi.univie.ac.at/cgi-bin/RNAWebSuite/RNAfold.cgi) to evaluate the impact of the 3’ UTR DRE changes on the edited gene.

### Function enrichment analysis

Gene ontology (GO) and Kyoko Encyclopedia of Genes and Genomes (KEGG) analysis were performed on the DAVID online platform (https://david.ncifcrf.gov/tools.jsp), and Enrichr (https://maayanlab.cloud/Enrichr/) (30). Items with a false discovery rate (FDR) < 0.05 were considered significant.

### Statistical analysis

RNA editing levels between V0 and COVID-19 vaccine groups were compared using the general linear model (GLM), and empirical *P*-values (*P*_GLM_) were calculated using the likelihood ratio test. Frequency data were analyzed using *Fisher*’s exact test. The correlation between RNA editing and gene expression levels was analyzed using the *Spearman* correlation method, and the correlation coefficient (*r*) and *P*-values were calculated accordingly.

## Results

### A-to-I RNA editing in blood sampled from COVID-19 vaccine recipients

**Figure 1:**
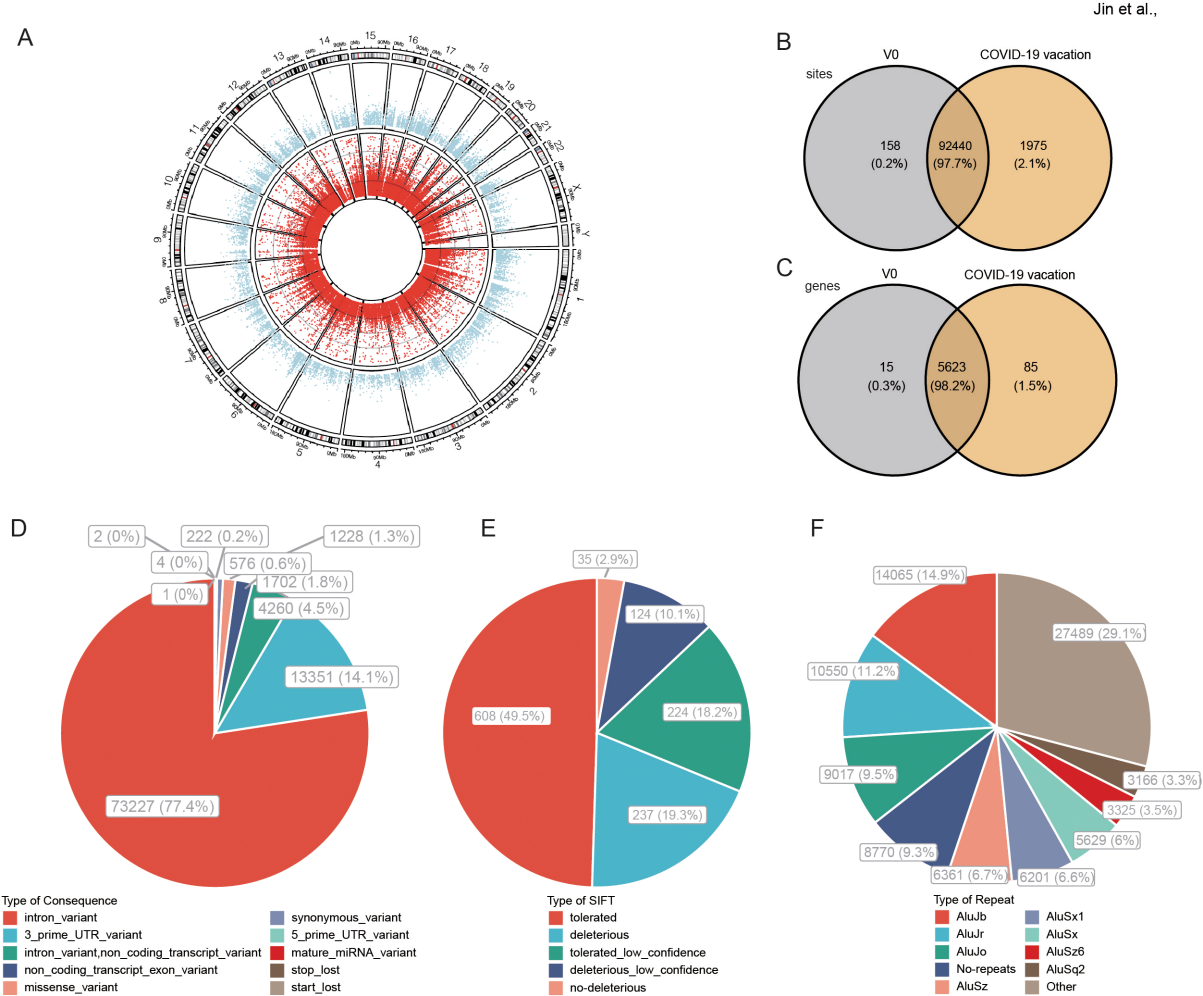
A-to-I RNA editing in response to COVID-19 vaccines identified from blood transcriptome cohort. **(A)** Circos plot of transcription gene expression (the outer circle) and A-to-I RNA editing sites (the inner circle) in the human brain. **(B, C)** The A-to-I RNA editing sites **(B)** and genes **(C)** of the two groups. **(D)** Functional types of variants resulted from A-to-I RNA editing. **(E)** About 29.4% of nonsynonymous variants are predicted by SIFT as possibly deleterious to the encoded proteins. **(F)** Most RNA editing sites are in *Alu* repetitive elements.

A total of 94573 high-confidence A-to-I RNA editing sites were observed in 5723 genes in all recipients’ blood (**Figure 1A**). The editing levels of these sites ranged from 1% to 100%, and were widely observed across all chromosomes. Among these RNA editing sites, 92440 (97.7%) were shared by two groups, 158 (0.2%) and 1975 (2.1%) sites were uniquely detected in control (V0) and COVID-19 vaccines groups, respectively (**Figure 1B**). As for edited genes, 5623 (98.2%) were shared, 15 (0.3%) and 85 (1.5%) genes were uniquely edited in V0 and COVID-19 vaccines groups, respectively (**Figure 1C**). The functional categories of these RNA editing sites included 73227 (77.4%) intronic variants, 13351 (14.1%) 3’ -untranslated region (3’ UTR) variants, and 1228 (1.3%) missense variants (**Figure 1D**). Sorts intolerant from tolerant (SIFT) analysis predicted that 361 (29.4%) of these missense variants might have a potential functional impact on the encoded protein (**Figure 1E**). 61.6% of these edited sites overlapped with Alu repetitive elements (**Figure 1F**).

### Temporal dynamics of RNA editing in response to COVID-19 vaccines

**Figure 2:**
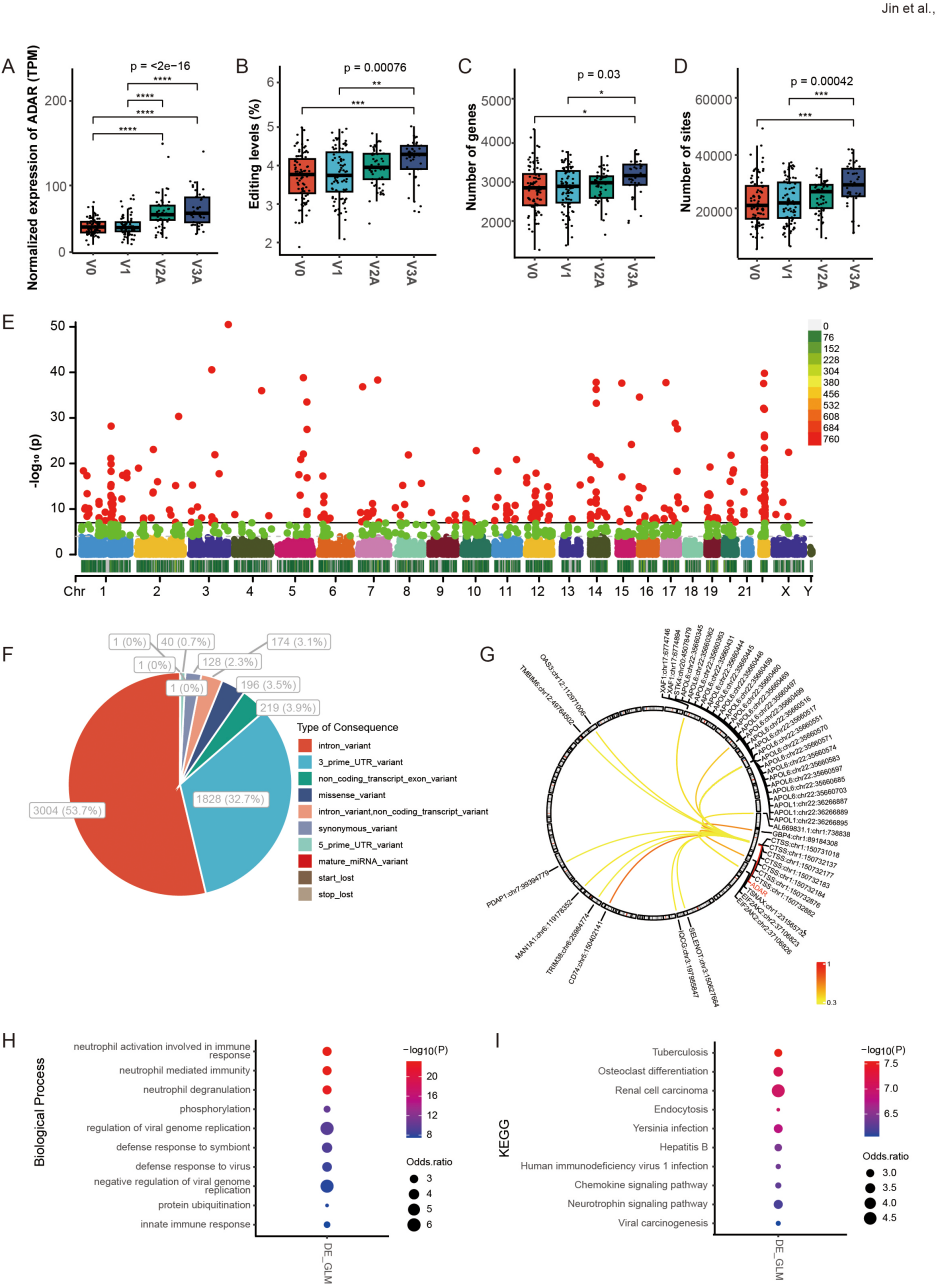
DRE A-to-I RNA editing in response to COVID-19 vaccines identified from blood transcriptome of three different doses of vaccination. **(A–D)** The *ADAR* expression and the average A-to-I RNA editing levels, as well as the number of edited genes and sites in four groups of individuals with different doses of vaccination (V0, V1, V2A, and V3A). **(E)** The Manhattan plot of DRE among the four groups. **(F)** The functional categories of DRE sites among the four groups. **(G)** The correlation between *ADAR* and the editing levels of individual DRE sites. **(H, I)** The items are shown for **(H)** biological processes and **(I)** the KEGG pathway in DRE genes in groups of different doses compared to V0. *P < 0.05; **P < 0.01; ***P < 0.001, ****P < 0.0001.

RNA editing was compared among the V0 group and vaccination groups that received three different doses of vaccine (V1, V2A, and V3A, as described in the original study). The results showed that *ADAR* expression and the average editing level, as well as the number of edited genes and editing sites, increased with vaccine doses, especially in V2A and V3A (**Figures 2A–D**). Among these RNA editing sites, 5592 differential RNA editing (DRE) sites were observed in 1820 genes across all chromosomes (**Figure 2E**, **Supplementary Table S1**). The functional categories of these DRE sites included 1828 (32.7%) 3’ UTR variants, 3004 (53.7%) intronic variants, and 219 (3.9%) non-coding transcript exonic variants (**Figure 2F**). Further analysis identified a subset of these DRE sites with their editing levels positively correlated with *ADAR* expression (|*r|* > 0.3, *p* < 0.05) (**Figure 2G**). A total of 1785 (98.1%) DRE genes contained two or more edited sites (**Supplementary Table S2**). The top ten genes ranked by the number of A-to-I RNA editing sites are shown in **Supplementary Table S3**. The top three genes were nicotinamide phosphoribosyltransferase (*NAMPT*), ring finger protein 213 (*RNF213*), and slingshot protein phosphatase 2 (*SSH2*).

### RNA editing in response to COVID-19 vaccines was mainly immune- and virus-related

Gene function enrichment showed that the differential blood RNA editing in response to COVID-19 vaccines was mainly involved in immune- and virus-related biological processes such as neutrophil activation involved in immune response, neutrophil-mediated immunity, neutrophil degranulation, and regulation of viral genome replication (**Figure 2H**); and KEGG pathways such as Yersinia infection, Hepatitis B, Human immunodeficiency virus 1 infection and Chemokine signaling pathway (**Figure 2I**).

### Recoded essential immune genes by missense RNA editing in response to COVID-19 vaccines

**Figure 3:**
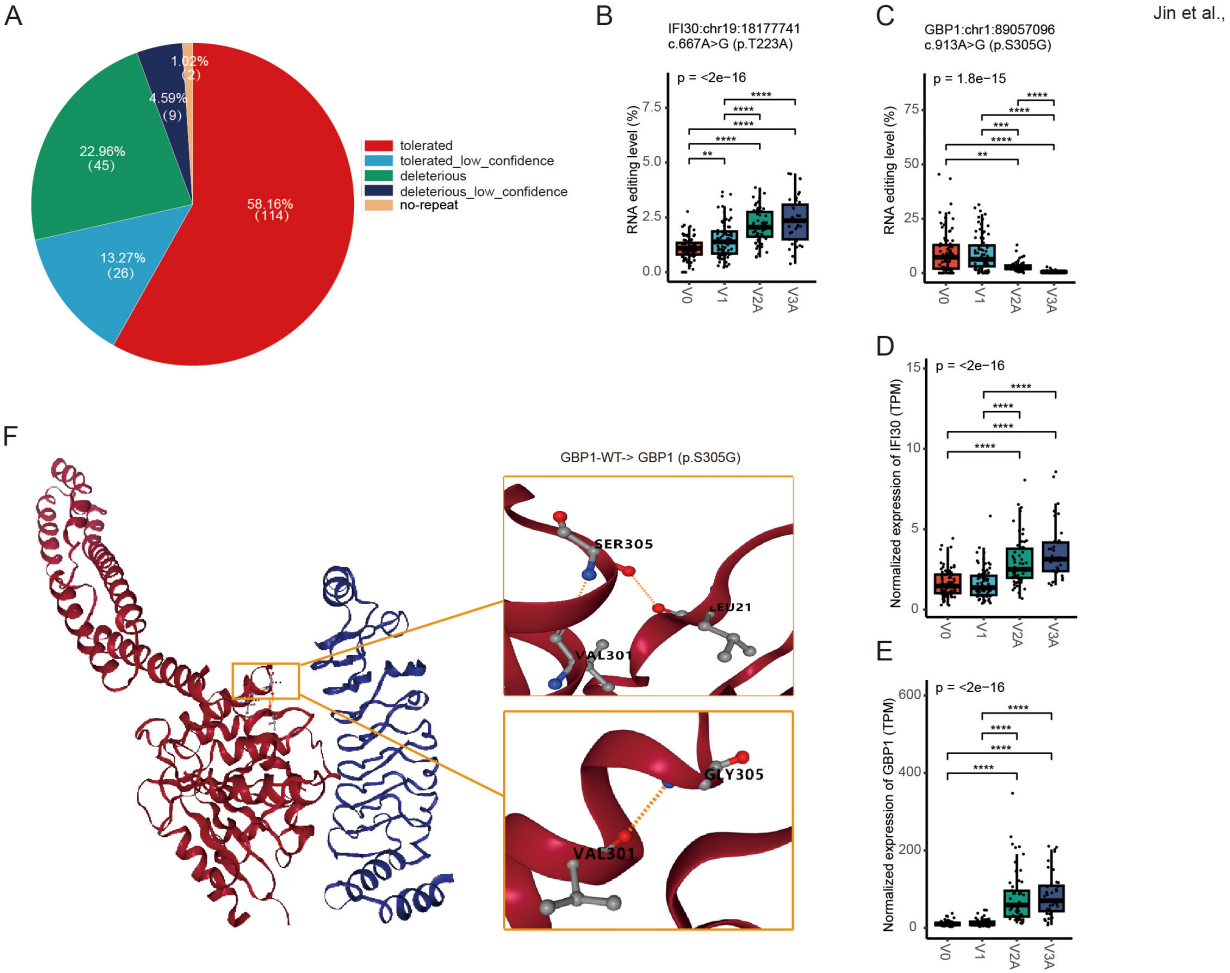
Missense DRE in *IFI30* and *GBP1*. **(A)** About 27.55% of missense variants are predicted by SIFT as possibly deleterious to the encoded proteins. **(B, C)** The site editing levels of *IFI30*:chr19:18177741 **(B)** and *GBP1*:chr1:89057096 **(C)**. **(D, E)** The gene expression levels of *IFI30* **(D)** and *GBP1* **(E)**. **(F)** Prediction of spatial structure of GBP1 protein. **P < 0.01; ***P < 0.001, ****P < 0.0001.

We then focused on missense RNA editing, which recodes the amino acid sequence of encoded proteins. 196 missense DRE sites in 140 genes were observed, with 27.55% predicted by SIFT to be deleterious or possibly deleterious to the encoded proteins (**Figure 3A**). Among the edited genes with the top ten missense DRE variants (**Supplementary Table S4**), two immune-related genes were observed, including IFI30 lysosomal thiol reductase (*IFI30*) and guanylate-binding protein 1 (*GBP1*). The missense RNA editing level of *IFI30* (*IFI30*:chr19:18177741, p.T223A) was significantly up-regulated (**Figure 3B**), whereas the *GBP1* missense editing (*GBP1*:chr1:89057096, p.S305G) was significantly down-regulated (**Figure 3C**), with the expression of both genes increased during COVID-19 vaccines (**Figures 3D, E**). The substitution of serine (S) with glycine (G) at codon 305 of *GBP1* was predicted to result in profound changes in its protein structure by DDMut (https://biosig.lab.uq.edu.au/ddmut/) (**Figure 3F**).

### 3’ UTR RNA editing regulation in response to COVID-19 vaccines could contribute to gene expression

**Figure 4:**
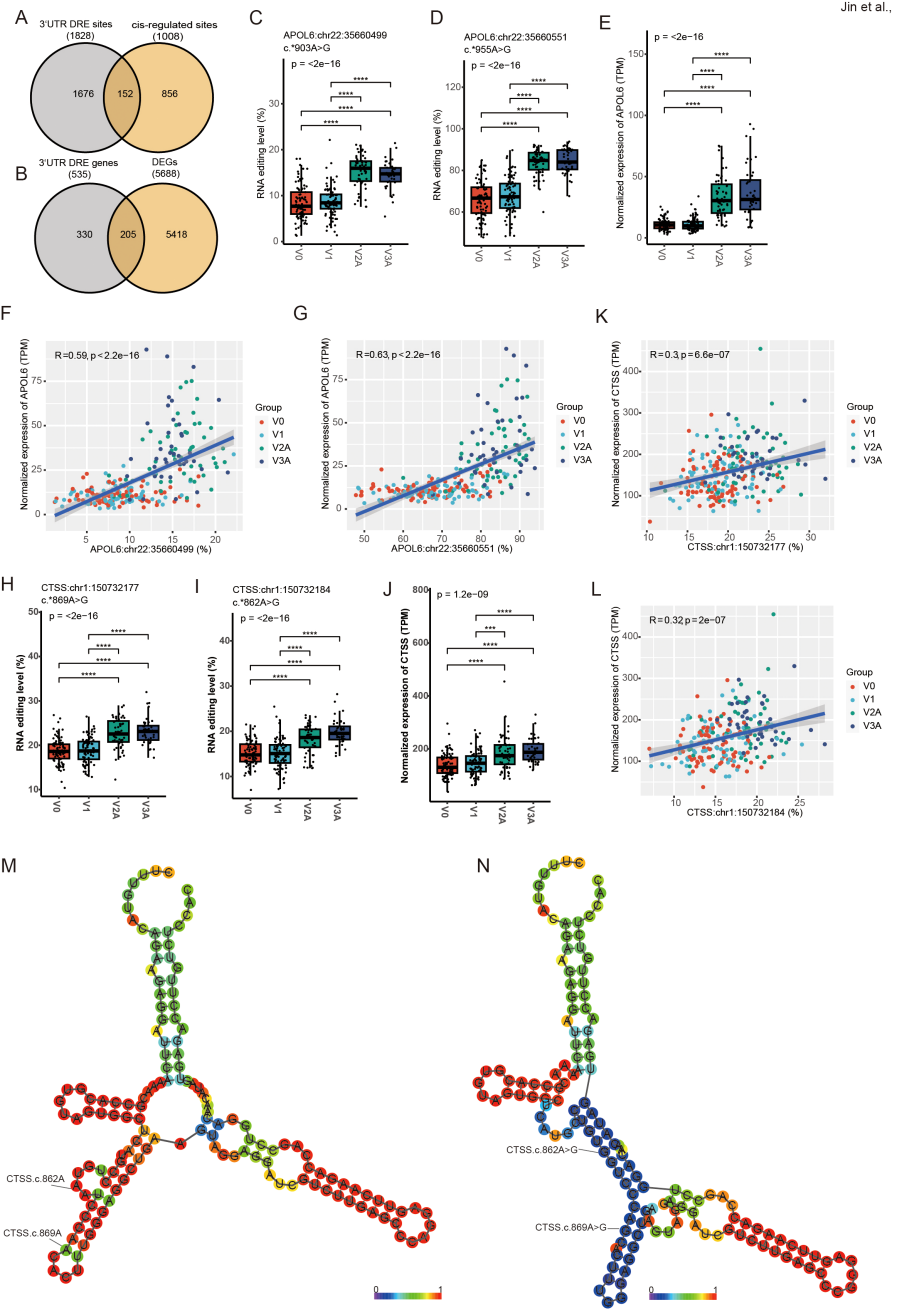
DRE in *APOL6* 3’ UTR and its potential *cis*-regulatory effects. **(A)** The Venn diagram of 3’ UTR DRE genes and *cis-*regulated genes. **(B)** The Venn diagram of 3’ UTR DRE genes and DEGs. **(C–E)** *APOL6*:chr22:35660499/35660551 editing level **(C, D)** and gene expression level **(E)**. **(F, G)** Correlation of *cis*-regulated *APOL6* gene expression level and editing sites (*APOL6*:chr22:35660499/35660551). **(H–J)** *CTSS*:chr1:150732177/150732184 RNA editing level **(H, I)** and gene expression level **(J)**. **(K, L)** Correlation of *cis*-regulated *CTSS* gene expression level and editing sites (*CTSS*:chr1:150732177/150732184). **(M, N)** The RNA secondary structure at the WT **(M)** and edited **(N)** of *CTSS*:chr1:150732177/150732184. ***P < 0.001, ****P < 0.0001.

3’ UTR editing has been reported to potentially be involved in regulating the expression of edited RNA. A total of 1828 3’ UTR DRE sites in 535 genes, with 152 (8.3%) predicted to *cis*-regulate 92 edited genes (**Figure 4A**, **Supplementary Table S5**), and 205 (38.3%) edited genes found differentially expressed (**Figure 4B**, **Supplementary Table S6**). The top ten 3’ UTR variants in our results are displayed in **Supplementary Table S7**. One of the most extensively differential editing was found in the apolipoproteins L6 (*APOL6*) gene. Both the RNA editing (*APOL6*:chr22:35660551, and *APOL6*:chr22:35660499) and gene expression levels of *APOL6* significantly increased upon COVID-19 vaccines (**Figures 4C–E**). *APOL6* 3’ UTR editing showed a strong positive correlation with its gene expression (**Figures 4F, G**).

As shown in **Figure 2G**, multiple editing sites in the cathepsin S (*CTSS*) 3’ UTR also showed strong correlations with ADAR expression. The results showed that both the RNA editing (*CTSS*:chr1:150732177, and *CTSS*:chr1:150732184) and gene expression levels of *CTSS* significantly increased upon COVID-19 vaccination (**Figures 4H–J**). *CTSS* 3’ UTR editing showed a strong positive correlation with its gene expression (**Figures 4K, L**) similar to that in *APOL6*. In addition, prediction of RNA secondary structure showed that both *CTSS* editing sites could significantly alter their RNA secondary structure (**Figures 4M, N**), which may contribute to the changes in *CTSS* expression.

### RNA editing in response to COVID-19 vaccines might influence RBP binding activity

**Figure 5:**
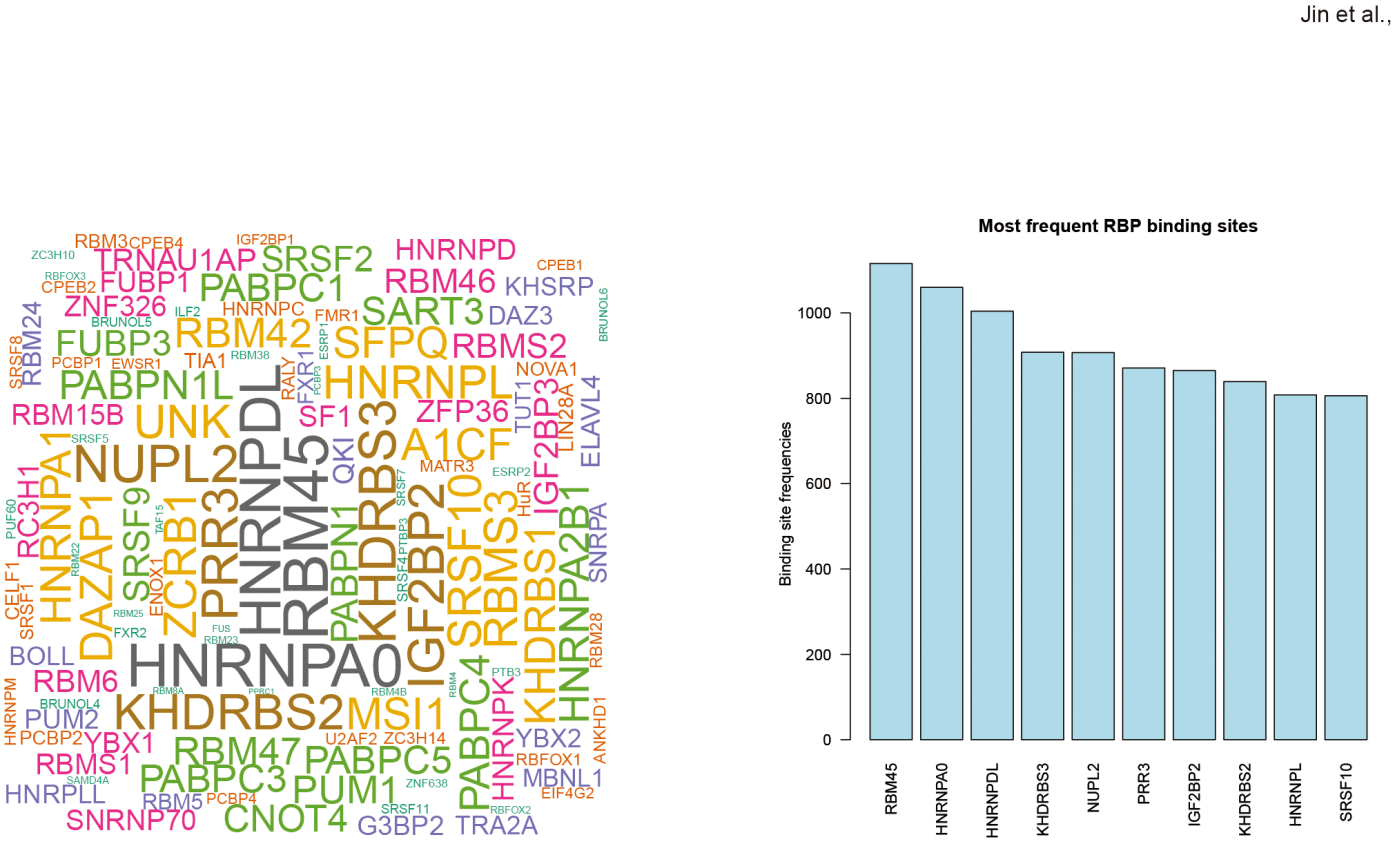
Differential RNA editing in response to COVID-19 vaccines might affect RBP binding sites. **(A)** Wordcloud plot of RBPs with binding sites overlapped with COVID-19 vaccines-associated DRE. **(B)** Top ten frequent RBPs with binding sites overlapped with differential RNA editing in response to COVID-19 vaccines.

To evaluate the potential effect of RNA editing on RBP binding, the RBPmap database was used to predict RBP binding sites that overlapped with DRE sites. The results in **Figure 5** showed the top RBPs ranked by their number of predicted overlapping DRE sites, with the top three as RNA-binding protein 45 (*RBM45*), heterogeneous nuclear ribonucleoprotein A0 (HNRNPA0), and heterogeneous nuclear ribonucleoprotein D-like (HNRNPDL).

### RNA editing in response to COVID-19 vaccines could be dose-dependent

**Figure 6:**
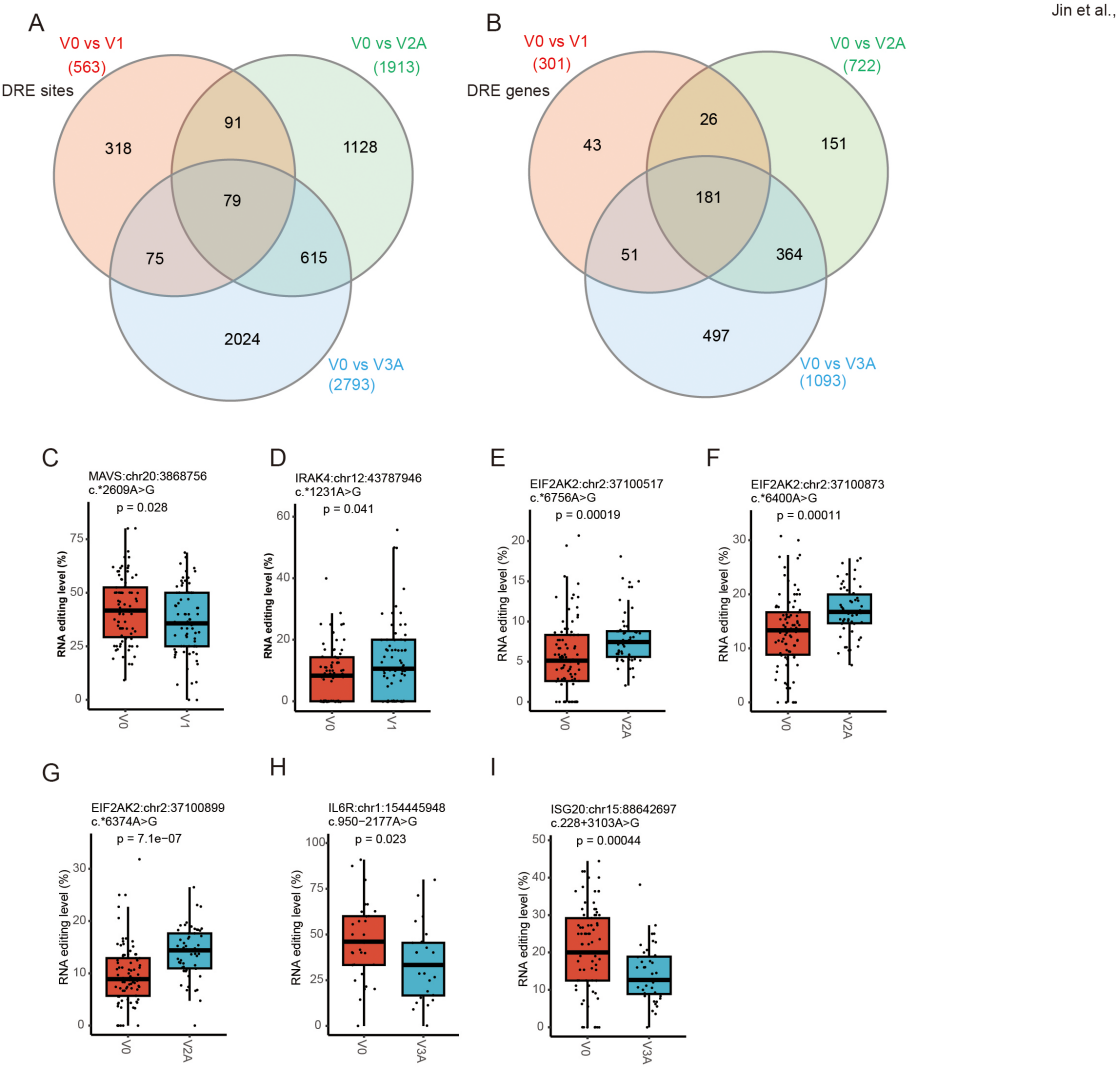
Dose-specific A-to-I RNA editing in response to COVID-19 vaccines. **(A, B)** The Venn diagram of four groups of DRE genes **(A)** and sites **(B)**. **(C, D)** The RNA editing levels of *MAVS* (chr20:3868756) **(C)** and *IRAK4* (chr12:43787946) **(D)** in V1. **(E, G)** The RNA editing levels of *EIF2AK2* (chr2:37100517/37100873/37100899) in V2A. **(H, I)** The RNA editing levels of *IL6R*:chr1:154445948 **(H)** and *ISG20*:chr15:88642697 **(I)** in V3A.

The three dose groups of COVID-19 vaccines were then compared to the V0 group separately to analyze DRE in individual dose groups (**Figure 6A**). Among the DRE sites in individual dose groups, 79 were shared by three vaccine dose groups, with 318, 1128, and 2024 exclusively found in V1, V2A, and V3A, respectively. As for genes differentially edited in individual dose groups, 181 were shared by the three vaccine dose groups, whereas 43, 151, and 497 were differentially edited exclusively in V1, V2A, and V3A, respectively (**Figure 6B**). Notably, such DRE in individual dose groups were found in several anti-viral- and immune-related genes, such as mitochondrial antiviral signaling protein *(MAVS*:chr20:3868756) and interleukin 1 receptor-associated kinase 4 (*IRAK4*:chr12:43787946) (**Figures 6C, D**) in V1, eukaryotic translation initiation factor 2 alpha kinase 2 (*EIF2AK2*:chr2:37100517/37100873/37100899) (**Figures 6E–G**) in V2A, and interleukin 6 receptor (*IL6R*:chr1:154445948) and interferon-stimulated exonuclease gene 20 (*ISG20*:chr15:88642697) (**Figures 6H, I**) in V3A.

**Figure 7:**
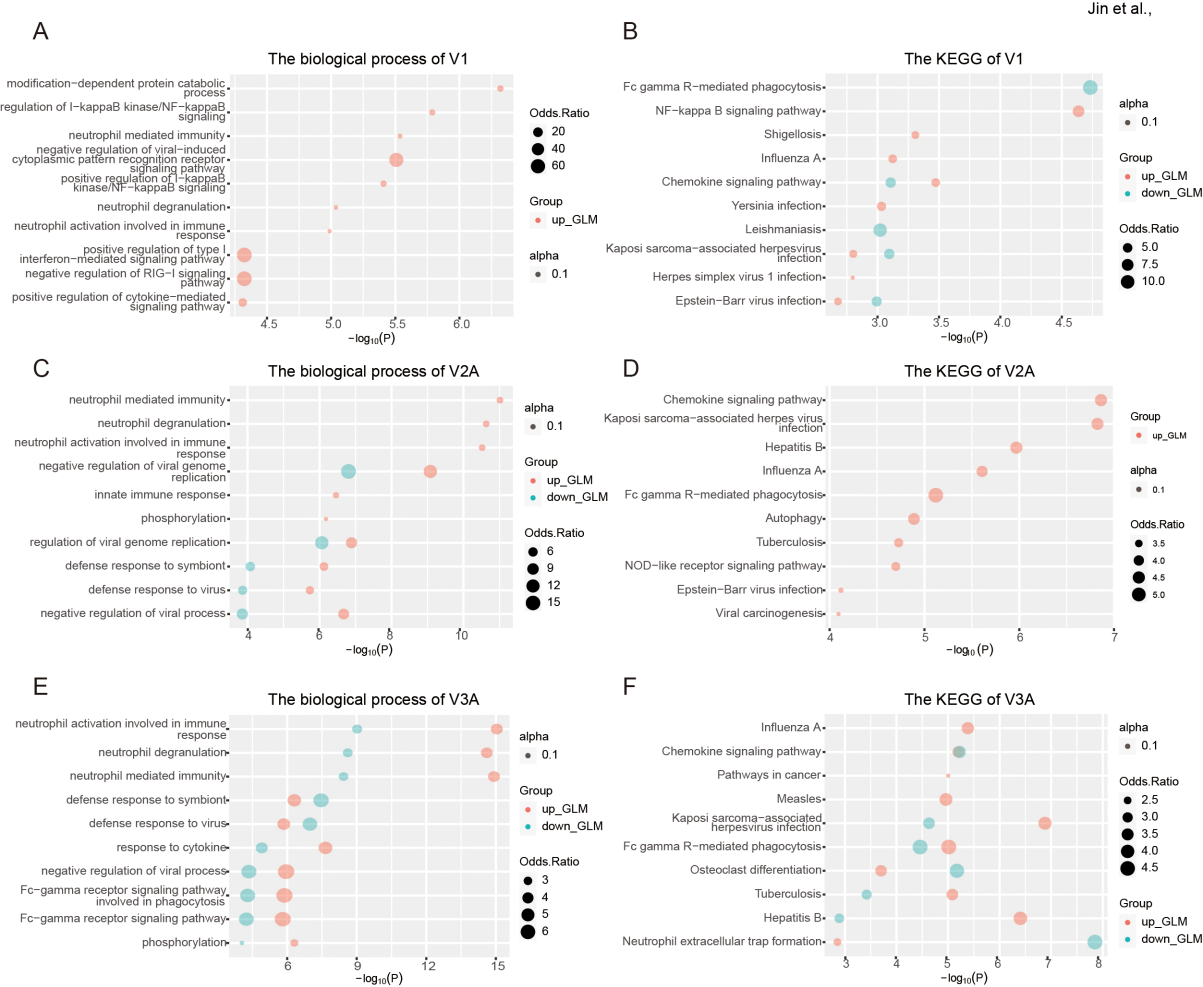
Functional relevance of differential RNA editing in response to COVID-19 vaccines in different individual dose groups. **(A, B)** The biological process **(A)** and KEGG pathways **(B)** of one-dose vaccination in response to one-dose vaccination. **(C, D)** The biological process **(C)** and KEGG pathways **(D)** of two-dose vaccination in response to two-dose vaccination. **(E, F)** The biological process **(E)** and KEGG pathways **(F)** of DRE in response to three-dose vaccination.

GO, and KEGG analysis was then performed to explore the biological function of such DRE in individual dose groups. In line with the results of overall DRE across the whole vaccination regime, DRE in different individual dose groups was also found to be mainly related to immune response and viral infections (**Figures 7A–F**). Notably, a set of gene functions and pathways, such as neutrophil-mediated immunity and defense response to virus, showed enrichment scores increasing with the number of vaccine doses, especially in V2A and V3A.

## Discussion

The underlying epigenetic changes and mechanisms related to RNA editing in response to COVID-19 vaccines remained largely unclear before our studies (31). By systematically investigating blood A-to-I RNA editing in response to COVID-19 vaccines, our results thus suggested a potentially important role of A-to-I RNA editing in regulating the immune response to COVID-19 vaccines.

Compared with V0, our results showed that *ADAR* expression and the average level of A-to-I RNA editing increased with doses during vaccination (**Figure 2**), pointing to enhanced RNA editing activity, an essential component of the anti-viral innate immune system. Vaccinated individuals could have enhanced immunity against COVID-19, which could also be in part attributed to the higher level of *ADAR* and RNA editing activity. Previous studies have shown that ADAR can inhibit hepatitis C virus (HCV) viral RNA replication by editing viral RNA during the virus replication (32). In addition to such direct anti-viral effects through RNA editing, ADAR might also influence the HCV viral cycle in an editing-independent manner by suppressing PKR activation (33, 34) and promoting up-regulated expression of anti-viral microRNAs (35). Emerging studies also reported that ADAR could modulate the immune response by editing host RNA during SARS-CoV-2 infection (20). Therefore, our results of RNA editing in response to COVID-19 vaccines showed a consistent role of ADAR-mediated A-to-I RNA editing in modulating the host’s immunity against SARS-CoV-2 infection. Moreover, individuals receiving more COVID-19 vaccine doses showed a higher immune response, in line with a higher level of RNA editing response that was also mainly related to immune and viral biological processes and pathways. Future research will further explore the underlying mechanism of specific editing events in the host’s antiviral immunity and their potential importance in the treatment and prevention of SARS-CoV-2 infections.

Missense RNA editing alters the amino acid sequences, potentially increasing protein diversity or affecting protein structure, stability, and functions (15). In our study, *IFI30* is a very significant missense DRE gene (**Supplementary Table S4**). *IFI30* encodes a crucial enzyme involved in antigen processing and presentation. Its expression could be induced by interferons, signaling molecules released by infected cells to alert neighboring cells of the presence of antigens (36). Additionally, IFI30 is associated with an enhanced immune and inflammatory response mediated by leukocytes and can regulate the IL6-STAT6 pathway (37). Increased IFI30 expression has been shown to enhance the ability of immune cells to eliminate various cancers by promoting the antigen presentation process (38–40). Recent studies have highlighted the therapeutic potential of targeting IFI30 in anti-tumor strategies, suggesting that modulating its expression or activity could regulate the immune response against tumor cells (37, 41). Moreover, IFI30 plays an important role in the initiation of CD4 and CD8 T-cell responses against viral peptides and exerts its antiviral effect by inhibiting cathepsin L activity of SARS-CoV, Ebola virus, and Lassa virus (42). In addition, GBP1 encodes a guanylate-binding protein that plays a key role in inflammatory pyroptosis and is involved in innate immunity against a diverse range of bacterial, viral, and protozoan pathogens (43, 44). Our analysis further suggested that these missense RNA editing events could exert their biological effects by altering the structure and function of the encoded proteins or *cis*-regulating the edited mRNA expression. Our findings thus pointed to a potentially important role of these missense editing in acting against viral infections by enhancing host immune and proinflammatory responses during COVID-19 vaccines.

Among the 3’ UTR DRE variants, the hyper-edited *APOL6* gene was one of the genes that exhibited the most significant differential RNA editing (**Supplementary Table S7**). Studies have revealed that APOL6 can inhibit the replication of certain viruses, such as coxsackie B virus and poliovirus (45), and its expression was significantly up-regulated in association with HIV-associated neurocognitive disorders (46). Furthermore, APOL6 are strongly upregulated upon inflammation via the Janus kinase (JAK)-signal transducer and activator of transcription (STAT) pathway, which are important signaling pathways involved in immune responses (47). Additionally, studies have shown that APOL6 is upregulated in immunotherapy responders, and enhances the efficacy of anti-tumor immunotherapy by promoting tumor cell apoptosis, necrosis, and pyroptosis pathways (48, 49). Therefore, our findings in APOL6 3’ UTR RNA editing were in line with the role of the gene in both antiviral defense and immune regulation. The *CTSS* gene was previously reported as a target of ADAR (50), strongly associated with type I IFN signature (51), and pivotal in MHC-II antigen loading and production of autoantibodies (52). Consistent with previous studies, elevated RNA editing levels of specific adenosines within *CTSS* 3’ UTR Alu elements correlate with increased *CTSS* expression (51). Moreover, the RNA editing levels of *CTSS* also significantly increased with vaccine dose (**Figures 4H, I**), and the changed editing sites also performed noticeable RNA structural alterations (**Figures 4M, N**). Therefore, the RNA secondary structure changes associated with RNA editing could also increased with vaccine doses. These findings underscore the potential vaccine dose-dependent RNA structure-specific editing and warranted future study on its functional implications.

Our results also indicate the possible involvement of RBP in the biological effects of RNA editing response to COVID-19 vaccines. NUPL2, also known as CG1, is required for the export of mRNAs containing poly(A) tails from the nucleus into the cytoplasm and could participate in the docking of viral Vpr at the nuclear envelope during HIV-1 infection (53). SART3 encoded an RNA-binding nuclear protein, which is found to be an important cellular factor for HIV-1 gene expression and viral replication. It also is transiently associated with U6 and U4/U6 snRNPs during the recycling phase of the spliceosome cycle and is involved in the regulation of mRNA splicing (54). Our findings thus warrant further experimental analysis of the actual biological impact of RBP binding in RNA editing response to COVID-19 vaccines.

While RNA transcription precedes RNA editing, it is well-established that RNA editing is a post-transcriptional modification mechanism that can dynamically modulate RNA stability, alternative splicing patterns, and even translation efficiency (55–57). These processes can significantly impact gene expression profiles in response to external stimuli such as vaccination. Specifically, RNA editing by ADAR enzymes can alter the sequence of transcripts by converting A to I, which are interpreted as G during translation. This editing process is known to affect the functional diversity of RNA molecules and consequently influence the expression of immune-regulatory genes. Furthermore, the temporal relationship between RNA transcription and editing underscores the dynamic nature of cellular responses to vaccine dosage. Changes in editing patterns may reflect adaptive responses of immune cells, where alterations in RNA editing profiles could fine-tune immune gene expression to optimize immune responses.

In conclusion, our study systematically investigated blood A-to-I RNA editing and revealed its dynamic response to COVID-19 vaccines. Our findings linked RNA editing with the immune response and antiviral effects of COVID-19 vaccines.

## Data Availability

The datasets presented in this study can be found in online repositories. The names of the repository/repositories and accession number(s) can be found in the article/**Supplementary Material**.

## References

[1] V'Kovski P Kratzel A Steiner S Stalder H Thiel V. Coronavirus biology and replication: Implications for Sars-Cov-2. Nat Rev Microbiol. (2021) 19:155–70. doi: 10.1038/s41579-020-00468-6 PMC7592455 33116300

[2] Ryan FJ Norton TS McCafferty C Blake SJ Stevens NE James J. A systems immunology study comparing innate and adaptive immune responses in adults to Covid-19 mrna and adenovirus vectored vaccines. Cell Rep Med. (2023) 4:100971. doi: 10.1016/j.xcrm.2023.100971 36871558 PMC9935276

[3] Skelly DT Harding AC Gilbert-Jaramillo J Knight ML Longet S Brown A. Two doses of Sars-Cov-2 vaccination induce robust immune responses to emerging Sars-Cov-2 variants of concern. Nat Commun. (2021) 12:5061. doi: 10.1038/s41467-021-25167-5 34404775 PMC8371089

[4] Bian S Li L Wang Z Cui L Xu Y Guan K. Allergic reactions after the administration of covid-19 vaccines. Front Public Health. (2022) 10:878081. doi: 10.3389/fpubh.2022.878081 35655467 PMC9152252

[5] Greenhawt M Abrams EM Shaker M Chu DK Khan D Akin C. The risk of allergic reaction to Sars-Cov-2 vaccines and recommended evaluation and management: A systematic review, meta-analysis, grade assessment, and international consensus approach. J Allergy Clin Immunol Pract. (2021) 9:3546–67. doi: 10.1016/j.jaip.2021.06.006 PMC8248554 34153517

[6] Gupta RK Topol EJ. Covid-19 vaccine breakthrough infections. Science. (2021) 374:1561–2. doi: 10.1126/science.abl8487 34941414

[7] Lipsitch M Krammer F Regev-Yochay G Lustig Y Balicer RD. Sars-Cov-2 breakthrough infections in vaccinated individuals: measurement, causes and impact. Nat Rev Immunol. (2022) 22:57–65. doi: 10.1038/s41577-021-00662-4 34876702 PMC8649989

[8] Chemaitelly H Abu-Raddad LJ. Waning effectiveness of Covid-19 vaccines. Lancet. (2022) 399:771–3. doi: 10.1016/s0140-6736(22)00277-x PMC8871492 35219385

[9] Goldberg Y Mandel M Bar-On YM Bodenheimer O Freedman L Haas EJ. Waning immunity after the bnt162b2 vaccine in Israel. N Engl J Med. (2021) 385:e85. doi: 10.1056/NEJMoa2114228 34706170 PMC8609604

[10] Gott JM Emeson RB. Functions and mechanisms of rna editing. Annu Rev Genet. (2000) 34:499–531. doi: 10.1146/annurev.genet.34.1.499 11092837

[11] Di Giorgio S Martignano F Torcia MG Mattiuz G Conticello SG. Evidence for host-dependent rna editing in the transcriptome of Sars-Cov-2. Sci Adv. (2020) 6:eabb5813. doi: 10.1126/sciadv.abb5813 32596474 PMC7299625

[12] Duan Y Tang X Lu J. Evolutionary driving forces of a-to-I editing in metazoans. Wiley Interdiscip Rev RNA. (2022) 13:e1666. doi: 10.1002/wrna.1666 33998151

[13] Emrani J Ahmed M Jeffers-Francis L Teleha JC Mowa N Newman RH. Sars-Cov-2, infection, transmission, transcription, translation, proteins, and treatment: A review. Int J Biol Macromol. (2021) 193:1249–73. doi: 10.1016/j.ijbiomac.2021.10.172 PMC8552795 34756970

[14] Heale BS Keegan LP McGurk L Michlewski G Brindle J Stanton CM. Editing independent effects of adars on the mirna/sirna pathways. EMBO J. (2009) 28:3145–56. doi: 10.1038/emboj.2009.244 PMC2735678 19713932

[15] Nishikura K. Functions and regulation of rna editing by adar deaminases. Annu Rev Biochem. (2010) 79:321–49. doi: 10.1146/annurev-biochem-060208-105251 PMC2953425 20192758

[16] Picardi E Mansi L Pesole G. Detection of a-to-I rna editing in Sars-Cov-2. Genes (Basel). (2021) 13(1):41. doi: 10.3390/genes13010041 35052382 PMC8774467

[17] Song Y He X Yang W Wu Y Cui J Tang T. Virus-specific editing identification approach reveals the landscape of a-to-I editing and its impacts on Sars-Cov-2 characteristics and evolution. Nucleic Acids Res. (2022) 50:2509–21. doi: 10.1093/nar/gkac120 PMC8934641 35234938

[18] Crooke PS 3rd Tossberg JT Porter KP Aune TM. Reduced a-to-I Editing of Endogenous Alu Rnas in Lung after Sars-Cov-2 Infection. Curr Res Immunol. (2021) 2:52–9. doi: 10.1016/j.crimmu.2021.04.001 PMC8084883 33969287

[19] Peng X Luo Y Li H Guo X Chen H Ji X. Rna editing increases the nucleotide diversity of Sars-Cov-2 in human host cells. PLoS Genet. (2022) 18:e1010130. doi: 10.1371/journal.pgen.1010130 35353808 PMC9000099

[20] Wei ZY Wang ZX Li JH Wen YS Gao D Xia SY. Host a-to-I rna editing signatures in intracellular bacterial and single-strand rna viral infections. Front Immunol. (2023) 14:1121096. doi: 10.3389/fimmu.2023.1121096 37081881 PMC10112020

[21] Tao J Ren CY Wei ZY Zhang F Xu J Chen JH. Transcriptome-wide identification of G-to-a rna editing in chronic social defeat stress mouse models. Front Genet. (2021) 12:680548. doi: 10.3389/fgene.2021.680548 34093668 PMC8173075

[22] Dobin A Gingeras TR. Mapping rna-seq reads with star. Curr Protoc Bioinf. (2015) 51:11.4.1–.4.9. doi: 10.1002/0471250953.bi1114s51 PMC4631051 26334920

[23] Li H Handsaker B Wysoker A Fennell T Ruan J Homer N. The sequence alignment/map format and samtools. Bioinformatics. (2009) 25:2078–9. doi: 10.1093/bioinformatics/btp352 PMC2723002 19505943

[24] Van der Auwera GA Carneiro MO Hartl C Poplin R Del Angel G Levy-Moonshine A. From fastq data to high confidence variant calls: The genome analysis toolkit best practices pipeline. Curr Protoc Bioinf. (2013) 43:11.0.1–.0.33. doi: 10.1002/0471250953.bi1110s43 PMC4243306 25431634

[25] Koboldt DC Zhang Q Larson DE Shen D McLellan MD Lin L. Varscan 2: somatic mutation and copy number alteration discovery in cancer by exome sequencing. Genome Res. (2012) 22:568–76. doi: 10.1101/gr.129684.111 PMC3290792 22300766

[26] McLaren W Gil L Hunt SE Riat HS Ritchie GR Thormann A. The ensembl variant effect predictor. Genome Biol. (2016) 17:122. doi: 10.1186/s13059-016-0974-4 27268795 PMC4893825

[27] Mansi L Tangaro MA Lo Giudice C Flati T Kopel E Schaffer AA. Rediportal: millions of novel a-to-I rna editing events from thousands of rnaseq experiments. Nucleic Acids Res. (2021) 49:D1012–d9. doi: 10.1093/nar/gkaa916 PMC7778987 33104797

[28] Liao Y Smyth GK Shi W. Featurecounts: an efficient general purpose program for assigning sequence reads to genomic features. Bioinformatics. (2014) 30:923–30. doi: 10.1093/bioinformatics/btt656 24227677

[29] Zhou Y Pan Q Pires DEV Rodrigues CHM Ascher DB. Ddmut: predicting effects of mutations on protein stability using deep learning. Nucleic Acids Res. (2023) 51:W122–w8. doi: 10.1093/nar/gkad472 PMC10320186 37283042

[30] Kuleshov MV Jones MR Rouillard AD Fernandez NF Duan Q Wang Z. Enrichr: A comprehensive gene set enrichment analysis web server 2016 update. Nucleic Acids Res. (2016) 44:W90–7. doi: 10.1093/nar/gkw377 PMC4987924 27141961

[31] Zhang Y Guo X Li C Kou Z Lin L Yao M. Transcriptome analysis of peripheral blood mononuclear cells in Sars-Cov-2 naïve and recovered individuals vaccinated with inactivated vaccine. Front Cell Infect Microbiol. (2021) 11:821828. doi: 10.3389/fcimb.2021.821828 35186784 PMC8851474

[32] Taylor DR Puig M Darnell ME Mihalik K Feinstone SM. New antiviral pathway that mediates hepatitis C virus replicon interferon sensitivity through adar1. J Virol. (2005) 79:6291–8. doi: 10.1128/jvi.79.10.6291-6298.2005 PMC1091666 15858013

[33] Garaigorta U Chisari FV. Hepatitis C virus blocks interferon effector function by inducing protein kinase R phosphorylation. Cell Host Microbe. (2009) 6:513–22. doi: 10.1016/j.chom.2009.11.004 PMC2905238 20006840

[34] Pfaller CK George CX Samuel CE. Adenosine deaminases acting on rna (Adars) and viral infections. Annu Rev Virol. (2021) 8:239–64. doi: 10.1146/annurev-virology-091919-065320 PMC12925331 33882257

[35] Liu G Ma X Wang Z Wakae K Yuan Y He Z. Adenosine deaminase acting on rna-1 (Adar1) inhibits hepatitis B virus (Hbv) replication by enhancing microrna-122 processing. J Biol Chem. (2019) 294:14043–54. doi: 10.1074/jbc.RA119.007970 PMC6755794 31366735

[36] Wang X Ge X Qin Y Liu D Chen C. Ifi30 is required for sprouting angiogenesis during caudal vein plexus formation in zebrafish. Front Physiol. (2022) 13:919579. doi: 10.3389/fphys.2022.919579 35910561 PMC9325957

[37] Zhu C Chen X Guan G Zou C Guo Q Cheng P. Ifi30 is a novel immune-related target with predicting value of prognosis and treatment response in glioblastoma. Onco Targets Ther. (2020) 13:1129–43. doi: 10.2147/ott.S237162 PMC7008640 32103982

[38] Buetow KH Meador LR Menon H Lu YK Brill J Cui H. High gilt expression and an active and intact mhc class ii antigen presentation pathway are associated with improved survival in melanoma. J Immunol. (2019) 203:2577–87. doi: 10.4049/jimmunol.1900476 PMC6832889 31591149

[39] Jiang W Zheng F Yao T Gong F Zheng W Yao N. Ifi30 as a prognostic biomarker and correlation with immune infiltrates in glioma. Ann Transl Med. (2021) 9:1686. doi: 10.21037/atm-21-5569 34988195 PMC8667103

[40] Zhou C Wei Z Zhang L Yang Z Liu Q. Systematically characterizing a-to-I rna editing neoantigens in cancer. Front Oncol. (2020) 10:593989. doi: 10.3389/fonc.2020.593989 33363023 PMC7758481

[41] Fan Y Wang X Li Y. Ifi30 expression predicts patient prognosis in breast cancer and dictates breast cancer cells proliferation via regulating autophagy. Int J Med Sci. (2021) 18:3342–52. doi: 10.7150/ijms.62870 PMC8364447 34400904

[42] Majdoul S Compton AA. Lessons in self-defence: inhibition of virus entry by intrinsic immunity. Nat Rev Immunol. (2022) 22:339–52. doi: 10.1038/s41577-021-00626-8 PMC8511856 34646033

[43] Ghosh A Praefcke GJ Renault L Wittinghofer A Herrmann C. How guanylate-binding proteins achieve assembly-stimulated processive cleavage of gtp to gmp. Nature. (2006) 440:101–4. doi: 10.1038/nature04510 16511497

[44] Li P Jiang W Yu Q Liu W Zhou P Li J. Ubiquitination and degradation of gbps by a shigella effector to suppress host defence. Nature. (2017) 551:378–83. doi: 10.1038/nature24467 29144452

[45] Kreit M Vertommen D Gillet L Michiels T. The interferon-inducible mouse apolipoprotein L9 and prohibitins cooperate to restrict theiler's virus replication. PLoS One. (2015) 10:e0133190. doi: 10.1371/journal.pone.0133190 26196674 PMC4510265

[46] Siangphoe U Archer KJ. Gene expression in hiv-associated neurocognitive disorders: A meta-analysis. J Acquir Immune Defic Syndr. (2015) 70:479–88. doi: 10.1097/qai.0000000000000800 26569176

[47] Adão R Guzik TJ. Inside the heart of Covid-19. Cardiovasc Res. (2020) 116:e59–61. doi: 10.1093/cvr/cvaa086 PMC7184380 32266937

[48] Liu K Chen Y Li B Li Y Liang X Lin H. Upregulation of apolipoprotein L6 improves tumor immunotherapy by inducing immunogenic cell death. Biomolecules. (2023) 13(3):415. doi: 10.3390/biom13030415 36979348 PMC10046184

[49] Liu Z Lu H Jiang Z Pastuszyn A Hu CA. Apolipoprotein L6, a novel proapoptotic bcl-2 homology 3-only protein, induces mitochondria-mediated apoptosis in cancer cells. Mol Cancer Res. (2005) 3:21–31. 15671246

[50] Stellos K Gatsiou A Stamatelopoulos K Perisic Matic L John D Lunella FF. Adenosine-to-inosine rna editing controls cathepsin S expression in atherosclerosis by enabling hur-mediated post-transcriptional regulation. Nat Med. (2016) 22:1140–50. doi: 10.1038/nm.4172 27595325

[51] Vlachogiannis NI Tual-Chalot S Zormpas E Bonini F Ntouros PA Pappa M. Adenosine-to-inosine rna editing contributes to type I interferon responses in systemic sclerosis. J Autoimmun. (2021) 125:102755. doi: 10.1016/j.jaut.2021.102755 34857436 PMC8713031

[52] Riese RJ Wolf PR Brömme D Natkin LR Villadangos JA Ploegh HL. Essential role for cathepsin S in mhc class ii-associated invariant chain processing and peptide loading. Immunity. (1996) 4:357–66. doi: 10.1016/s1074-7613(00)80249-6 8612130

[53] Le Rouzic E Mousnier A Rustum C Stutz F Hallberg E Dargemont C. Docking of hiv-1 vpr to the nuclear envelope is mediated by the interaction with the nucleoporin hcg1. J Biol Chem. (2002) 277:45091–8. doi: 10.1074/jbc.M207439200 12228227

[54] Bell M Schreiner S Damianov A Reddy R Bindereif A. P110, a novel human U6 snrnp protein and U4/U6 snrnp recycling factor. EMBO J. (2002) 21:2724–35. doi: 10.1093/emboj/21.11.2724 PMC126028 12032085

[55] Hsiao YE Bahn JH Yang Y Lin X Tran S Yang EW. Rna editing in nascent rna affects pre-mrna splicing. Genome Res. (2018) 28:812–23. doi: 10.1101/gr.231209.117 PMC5991522 29724793

[56] Rieder LE Reenan RA. The intricate relationship between rna structure, editing, and splicing. Semin Cell Dev Biol. (2012) 23:281–8. doi: 10.1016/j.semcdb.2011.11.004 22178616

[57] Booth BJ Nourreddine S Katrekar D Savva Y Bose D Long TJ. Rna editing: expanding the potential of rna therapeutics. Mol Ther. (2023) 31:1533–49. doi: 10.1016/j.ymthe.2023.01.005 PMC9824937 36620962

